# A Web-Based Positive Psychology App for Patients With Bipolar Disorder: Development Study

**DOI:** 10.2196/39476

**Published:** 2022-09-19

**Authors:** Bart Geerling, Saskia M Kelders, Anja W M M Stevens, Ralph W Kupka, Ernst T Bohlmeijer

**Affiliations:** 1 Department of Psychology, Health and Technology Centre for eHealth and Wellbeing Research University of Twente Enschede Netherlands; 2 Centre for Bipolar Disorder Dimence Mental Health Institute Deventer Netherlands; 3 Optentia Research Focus Area North-West University Vanderbijlpark South Africa; 4 Department of Psychiatry Mental Health Program, Amsterdam Public Health Amsterdam UMC location Vrije Universiteit Amsterdam Amsterdam Netherlands

**Keywords:** bipolar disorder, positive psychology, cocreation, mobile health, mHealth, web-based, psychology, bipolar, intervention, quality of life, mental illness, pilot, self-esteem, acceptance, social isolation, manic episode, manic, self-help, positive, mobile phone

## Abstract

**Background:**

Patients with bipolar disorder (BD) report lower quality of life and lower levels of well-being than the general population. Despite the growing availability of psychotherapeutic and self-management interventions, important unmet needs remain. These unmet needs are closely linked to positive psychology domains. Although a growing number of studies have evaluated the impact of positive psychology interventions (PPIs) on patients with severe mental illness in general, only few have addressed the application of positive psychology for BD.

**Objective:**

This study aimed to gain insight into the opinions of patients with BD and health care professionals about (web-based) PPIs for BD and to develop and pilot-test an app containing PPIs specifically designed for patients with BD.

**Methods:**

The study was conducted in accordance with the Center for eHealth and Disease Management road map principles and incorporated cocreation and designing for implementation. Data were collected using focus group discussions, questionnaires, rapid prototyping, and web-based feedback on a prototype from the participants. In total, 3 focus groups were conducted with 62% (8/13) of patients with BD and 38% (5/13) of professionals. The collected data were used to develop a smartphone app containing short PPIs. The content was based on PPIs for which a solid base of evidence is available. Finally, a pilot test was conducted to test the app.

**Results:**

Focus groups revealed that PPIs as part of the current BD treatment can potentially meet the following needs: offering hope, increasing self-esteem, expressing feelings, acceptance, and preventing social isolation. Some patients expressed concern that PPIs may provoke a manic or hypomanic episode by increasing positive affect. The pilot of the app showed that the PPIs are moderately to highly valued by the participants. There were no adverse effects such as increase in manic or hypomanic symptoms.

**Conclusions:**

With the systematic use of user involvement (patients and professionals) in all steps of the development process, we were able to create an app that can potentially fulfill some of the current unmet needs in the treatment of BD. We reached consensus among consumers and professionals about the potential benefits of PPIs to address the unmet needs of patients with BD. The use of PPI for BD is intriguing and can be usefully explored in further studies. We emphasize that more evaluation studies (quantitative and qualitative) that are focused on the effect of PPIs in the treatment of BD should be conducted. In addition, to establish the working mechanisms in BD, explorative, qualitative, designed studies are required to reveal whether PPIs can address unmet needs in BD.

## Introduction

Bipolar disorder (BD) is defined as a chronic mental illness with recurrent mood episodes, with manic, hypomanic, and depressive episodes alternating with euthymic periods. The illness mostly begins in adolescence and young adulthood [1]. It is estimated that BD I and II disorders occur in 2% of the world’s population, and another estimated 2% has a subthreshold BD [2]. In the Netherlands, the prevalence of BD in the adult population is 1.3% [3].

Patients and their significant others face significant burden when confronted with BD. Owing to the early onset, severity, and chronicity, BD is a potentially disabling illness [4-6]. Even during euthymic periods between episodes, the illness may lead to impairment and significant burden [7]. Factors contributing to the burden are persistent subsyndromal mood symptoms, stigmatization, cognitive impairment, comorbid conditions, and side effects of pharmacotherapy [7]. The burden of BD may increase over the longitudinal course of the disease. Illness progression has been described using various staging models for BD [8], and it is expected that the burden of illness is more prominent in the later stages with multiple recurrences or persistent unremitting illness [9]. Persistence of mood symptoms between episodes is a significant predictor of depression and functional impairment [10]. The level of (subsyndromal) depressive symptoms correlates positively with the degree of functional impairment [11] and, therefore, with a high burden and low quality of life. Despite the growing availability of psychotherapeutic and self-management interventions, important unmet needs remain, including those that are not directly related to mood symptoms in patients with BD.

Previous studies have established that there are major unmet needs in the management of BD [12-17]. Needs are defined as what people “desire to receive from healthcare services to improve overall health” [18]. The most common needs during depression and mania or hypomania can be summarized as encouragement to seek effective (pharmacological) treatment to reduce symptoms. During remission and subsyndromal episodes, there is a need for treatment that prevents future episodes and a need for easily available psychosocial interventions [13,16].

Most common needs are satisfied to some degree during regular treatment. Some studies have categorized additional unmet needs addressing social and psychological functioning using questionnaires (Need for Care Questionnaire or self-developed questionnaires) [14,15]. In social functioning, support with loneliness, grief counseling, acceptation, social isolation, and coping with others are frequently mentioned [14,15,17]. Hope, expressing feelings, and increasing self-confidence are mentioned as unmet needs in psychological functioning [12,14-17]. These needs are closely linked to the domain of personal recovery [19]. The similarity lies in the aim to apply interventions to increase mental well-being, rather than just symptomatic recovery. In personal recovery, there are 5 recognizable components: connectedness, hope, identity, meaning, and empowerment, indicated by the CHIME acronym [19]. Bird et al [20] added 3 components (practical support, issues around diagnosis and medication, and skepticism surrounding recovery) to CHIME to address the needs of those who are in an early stage of recovery. Several interventions have been developed based on the CHIME framework, focusing on increasing hope, strengths, connectedness, and empowerment [21,22]. A potential treatment approach to improve personal recovery and address the unmet needs of patients with BD is positive psychology (PP) [14,23-25].

PP is a relatively new field in psychology that focuses on improving positive feelings, behaviors, and cognitions [26-28]. Some important evidence-based types of PP interventions (PPIs) are *savoring* [29-32], *practicing kindness* [33], *experiencing and expressing gratitude* [34-38], *creating meaning and goal setting* [37,39-43], *positive relations* [23,37,44,45], and *using personal strengths* [36]. Meta-analyses have found that PPIs have small to moderately significant effects on well-being and distress in general populations [28,46-48] and clinical populations [49,50]. Some small studies have also shown promising effects of PPIs on the mental health of patients with BD [51-53]. Recently, a fully powered trial was conducted to evaluate the impact of a positive psychotherapy group treatment on the mental well-being of patients with BD in comparison with treatment as usual [54,55], reporting promising medium to large between-group effects, with sustained effect after 6 months [55]. Applying PPIs in mental health care fits well in a recently developed model of sustainable mental health [56]. In this model, both mental illness and mental well-being are proposed as vital outcomes in psychiatry. A core aim of treatment is to promote the patients’ ability to adapt. This ability is hindered by barriers such as dysfunctional biological and psychological processes and enhanced by resources such as positive emotions, hope, meaning, and positive relationships. Bohlmeijer and Westerhof [57] argue that there is a need for balanced mental health care. PPIs primarily target the development of resources that support the patient with personal recovery and maintaining mental health.

Digital health interventions are increasingly common in mental health treatment. In PP, eHealth interventions are known as online PPIs (OPPIs). Studies in this area have shown that OPPIs can enhance well-being and reduce depressive symptoms [58]. Results from recent studies show a significant effect of OPPIs [59-61]. For those individuals who found that the interventions are relevant for their symptoms, OPPIs seem more acceptable [62]. It has also been found that the adjustability of a digital application improves its acceptability [62,63]. Patients with depressive symptoms seem to benefit more from OPPIs [64]. These findings suggest that digital applications can be a promising way to implement PPIs for patients with (bipolar) depression or those with low well-being levels.

Potential benefits of digital health interventions lie in the improved accessibility, flexibility in both standardization and personalization, interactivity, and consumer engagement [65]. Successful implementation of eHealth applications requires careful consideration of individual needs and cocreation with key stakeholders in both the design and implementation phases. However, adoption by users and professionals is not always easily achieved; professionals can be skeptical about the potential benefits and experience little support in using eHealth applications [66]. Implementing a web-based recovery treatment program for patients with severe mental illness revealed that they were not easily engaged [67]. However, these challenges should not prevent a push forward for health care technology changes [68]. Therefore, development and implementation of technical innovations require thorough communication and coordination between health care professionals and patients. In developing eHealth applications, user involvement is essential for technology adoption and use, increased user satisfaction, trust, and usability and is needed for successful implementation [69]. To achieve engagement, commitment, confidence, and a more positive attitude toward new eHealth interventions from potential users, van Gemert-Pijnen et al [70] developed a holistic approach for designing and implementing eHealth applications.

In summary, the overall burden of BD is vast and significantly impairs patients’ quality of life. There are important unmet needs for patients with BD, which are mainly related to personal recovery. PP is a promising treatment approach to improve personal recovery. Implementing PPIs in digital interventions is potentially empowering and cost-effective. However, systematic user involvement in digital health interventions is vital. This study aimed to gain insight into the opinions of patients with BD and health care professionals about (web-based) PPIs for BD and to develop and pilot-test an app containing PPIs specifically designed for patients with BD.

The following stages were addressed in the study:

1. Assessment of opinions in Focus group meetings (contextual inquiry)
2. Assessment of preferences and requirements (value specification [VS] and design)
3. Assessment of use and satisfaction with positive psychology app and interventions (operationalization)

## Methods

### Design

The study focused on gathering information from patients and professionals about the use of PP to develop an app containing PPIs for patients with BD. A qualitative design was used to identify and characterize opinions and needs. The target group comprised patients with BD and professionals (end users). To enable a broad perspective on the potential benefits of web-based PPIs for patients with BD, focus group discussions (FGDs), paper prototyping (PPT), and pilot test (PT) were used. The method was modeled on the principles of the holistic development approach described in the Center for eHealth and Disease Management (CeHRes) road map, for example, the participatory process and continuous evaluation cycles [70]. Our study covered the first 3 steps of the model: contextual inquiry, VS, and design. The CeHRes aims to facilitate continuing process of evaluation and participation of all stakeholders.

### Ethics Approval

Ethics approval was obtained from the University of Twente (18067) and the scientific board of the Dimence Mental Health Institute, where the study was conducted. All participants signed an informed consent form, in which they also agreed to be audio recorded during the focus groups (FGs).

### Participants

Participants were patients with BD I or II in a euthymic episode and professionals treating patients with BD who were in possession of a smartphone and were willing to travel to attend the FGs. They participated in all 3 steps of the development process. When recruiting our sample, we aimed for maximum variation among the participants of the FG. Therefore, we used several recruitment strategies. Patients were recruited from an outpatient clinic and the national advocacy group (Plusminus) to receive input from different regions of the Netherlands. In using this method of recruiting, we tried to avoid selection bias. All the patients received treatment as usual for BD. A call for participation was posted in the advocacy group’s magazine. The researchers who participated in the FGs (BG and SK) were not involved in the participants’ treatments. Professionals in the FG were involved in various disciplines (psychiatrists, psychiatric nurses, and psychologists) and recruited from the Dutch Foundation for Bipolar Disorders (Kenniscentrum Bipolaire Stoornissen), a chapter of the International Society for Bipolar Disorders.

Participants contributed to all study phases (FG, PPT, and PT); however, not all participants attended every FG. None of the participants in the FG had professional relationships, which means that none of the patients were treated by the professionals or otherwise involved with each other.

### Procedure and Materials

#### Overview

In total, three 2-hour FGDs were conducted in September 2018 and October 2018. At the beginning of the FG, a clear statement was made about confidentiality to ensure that the participants can independently provide their opinion. The sessions were semistructured, with the use of a topic list. In the first FGD, we explored experiences with PP. In the second FGD, we gathered the requirements for a potential PP app and the needs that will be covered by a PPI app. In the third FGD, the final requirements for the PPI app for BD were established. Each FG was briefly introduced with a PowerPoint (Microsoft Corp) presentation to reveal the purpose of that discussion. The participants were informed about the topics before the FG. Table 1 provides an overview of the techniques used during the FGs.

**Table 1:** Overview of techniques used during the focus groups.

FGD^a^	Brown paper exercise	Paper prototyping	Rapid prototyping test	Valuation PP^b^ exercises
1	✓			
2	✓	✓		✓
3			✓	

^a^FGD: focus group discussion.

^b^PP: positive psychology.

#### Assessment of Opinions in FMGs (Contextual Inquiry)

After the brief introduction to the first FG, participants were asked to write down the experiences with PP, needs that PP could address, and requirements of a PPI app on post-it memos collected on flip charts (brown paper exercise). These statements were the basic assumptions used to initiate the discussion about the various opinions. The agreements and disagreements were summarized, and in the next FG, the members were asked again to mention a preference. This process was repeated until consensus was reached. The discussions were recorded and transcribed, followed by a consensus check. After each session, a report was made and sent to the participants for validation and comments to increase objectivity (member checking). Concepts of a *consensus document* were discussed in the second and third meetings and established after the last session.

#### Assessment of Preferences and Requirements (VS and Design)

We also used the PPT method in FG 2 to test the preferences. On the basis of the preferences mentioned in the FG, we created a PP exercise (being grateful). The participants rated screenshots of the exercise in terms of content, wording, and design. Subsequently, we asked the participants their opinions and valuations about different PPIs to establish which PPIs seemed suitable for patients with BD. The 6 categories of PPI’s (positive emotions, resilience, positive relations, optimism and hope, self-compassion, and strengths) were explained and practiced with 1 exercise per category. Then, the participants scored each type in a positive or negative appreciation. We conducted a rapid prototyping (RPT) test to establish the participants’ opinions about a possible web-based PPI. On the basis of the first FG input, we built an exercise in ‘The Incredible Intervention Machine,’ an app specially designed to perform prototyping and pilot-testing of newly developed apps in a research setting [71].

On the basis of the results of the FGs, an app with PPIs (Well-being Bipolar Disorder) was designed. Then, this app was evaluated in a PT.

#### Assessment of Use and Satisfaction With PP App and Interventions (Operationalization)

After the development process, we tested our app to evaluate whether the results of the previous steps of the development process had been implemented satisfactorily. Then, we maximized user involvement. In the PT, we tested the intervention app containing 7 PPI exercises in 1 week. The participants were asked to perform 1 exercise daily. After they completed 1 exercise, the following exercise appeared the next day. We collected data on the exercises separately through ranking after every exercise and the possibility to provide remarks about the exercise. We also collected data on the use of the app (preferences in settings, frequency of use, and number of completed exercises). After completing all the exercises, a final evaluation within the app was conducted to establish experiences about the intervention.

### Data Analysis

ATLAS.ti7 was used for the analysis of the data from the FG. The Colaizzi method, as described by Shosha [72], was used to process the data. The FG recordings were transcribed verbatim for the analysis in 3 phases: open coding, axial coding, and selective coding. Researcher triangulation was used to increase the objectivity of data analysis. The app’s quantitative data (rating of the exercises) were collected, and average scores for each exercise and all the 7 exercises were calculated using SSPS. In addition, we also calculated the average scores of the differences between professionals and patients. The qualitative data (open answers in the evaluation module) were collected and analyzed through inductive coding.

## Results

### Participants

For the study, 17 participants (n=11, 65% patients and n=6, 35% professionals) were recruited. In total, 24% (4/17) of the participants withdrew (3/4, 75% patients and 1/4, 25% professional) before the start of the study owing to personal reasons or because they were not available at the time of the FGs. For the last phase (ie, PT of the app), 6 participants were added to broaden the input with opinions of participants who did not participate in the FGs. Demographics are shown in Table 2. Table 3 shows the participation in the FG, RPT, and PT.

**Table 2:** Demographics of participants of the focus groups.

Characteristics		Total (N=13), n (%)	Patients (n=8), n %)	Professionals, (n=5), n (%)
**Age (years)**				
	16-24	1 (8)	1 (13)	0 (0)
	25-40	4 (31)	3 (38)	1 (20)
	41-55	5 (38)	2 (25)	3 (60)
	56-70	3 (23)	2 (25)	1 (20)
Sex (female)		7 (54)	4 (50)	3 (60)
**Marital status**				
	Single	2 (15)	2 (25)	0 (0)
	In relationship	6 (46)	4 (50)	2 (40)
	In relationship and has children	5 (38)	2 (25)	3 (60)
**Education**				
	Primary school	0 (0)	0 (0)	0 (0)
	High school	3 (23)	3 (38)	0 (0)
	Higher professional education	8 (62)	5 (63)	3 (60)
	University	2 (15)	0 (0)	2 (40)

**Table 3:** Participation in the different stages of the study (N=19).

Part of the study	Patients (n=10), n (%)	Professionals (n=9), n (%)	Total (n=19), n (%)
FG^a^ 1	4 (21)	2 (11)	6 (32)
FG 2	4 (21)	3 (16)	7 (37)
FG 3	4 (21)	4 (21)	8 (42)
PPT^b^	4 (21)	3 (16)	7 (37)
RPT^c^	4 (21)	4 (21)	8 (42)
PT^d^	10 (53)	9 (47)	19 (100)

^a^FG: focus group.

^b^PPT: paper prototyping.

^c^RPT: rapid prototyping.

^d^PT: pilot test.

### Focus Group Discussions

The results of all FGs are presented based on the different stages of the CeHRes road map. The results of the contextual inquiry and VS are summarized to provide a good overview of the FG results.

### Assessment of Opinions in FMGs (Contextual Inquiry)

In the FG, first, we discussed the participants’ level of experience with PP. Second, we created an inventory of expected advantages and disadvantages when PPI is applied for BD. The results are summarized in Table 4.

Among the FG members, there was little experience with PP, as shown in Table 4. The participants did not have experiences with specific evidence-based PPIs. The experiences can be found in adjoining therapeutic areas (eg, mindfulness) or more personal PP solutions (eg, recognizing positive moments or thinking about possibilities rather than about problems). The FG members mentioned possible advantages: focusing on small steps, making positive pictures or movies, monitoring positive feelings, and giving themselves a positive message. Unmet needs such as hope, acceptance, and increasing self-confidence appear to be the most promising ones that PPIs may address in BD. The participants also mentioned various potential disadvantages of PPIs in 2 categories: illness-related and personal factors. The expectation of disadvantages regarding their illness is seen in both mania and depressive episodes. The participants did not expect benefits from PPIs in severe depressive episodes, or even the inability to perform the exercises during severe depression, leading to disappointment rather than satisfaction. They also foresaw further mood dysregulation toward manic stages when they are already hypomanic owing to exercises that stimulate positive emotions, feelings, or happiness. On a personal level, the participants fear forced positive statements that are not consistent with their self-esteem and the risk that PPIs can push the goal setting level very far (perfectionism). For both mentioned domains, they fear a possible counterproductive outcome when PPIs are applied.

**Table 4:** Contextual inquiry, experiences, and expectations.

Experiences	Number of times initially mentioned in brown paper exercise^a^	Expectations (advantage)	Unmet needs, as described in the literature [12,14-17]	Expectation’s disadvantage	Label
Make mantras for yourself	1×	Avoid stigmatization	Social isolation and acceptation	Forced positive statements	Personal level
Exercising mindfulness	2×	Helpful in “gloomy” periods	Hope	Not beneficial when severely depressed	Illness-related factor
Caring for others	1×	Monitoring of positive feelings	Grief counseling and acceptation	Possibility of high goal setting	Personal level
Writing a “stoic journal” daily	1×	Focusing on small steps (near future)	Hope	Fear that positive feeling can lead to hypomania or mania	Illness-related factor
Knowing through reading about PP^b^	1×	Express gratitude	Expressing feelings	—^c^	—
Recognize positive moments	1×	Positive messages to yourself	Increasing self-confidence and hope	—	—
User involvement as a positive activity	1×	Create positive daily pictures or movies	Increasing self-confidence and hope	—	—
Thinking in possibilities	2×	—	—	—	—

^a^Participants wrote their thoughts and opinions on memo blocks sheets before the discussion started.

^b^PP: positive psychology.

^c^Not available.

### Assessment of Preferences and Requirements (VS)

The aim of the FGs was to address opinions about the use and requirements of an app. First, the participants were asked about their opinions on the different categories of PPIs (Table 5). The FG participants were unanimous in the appreciation of the exercises in positive emotions and resilience categories and found positive relations and strengths to be applicable for patients with BD. In the categories of self-compassion, hope, and optimism, the FG participants were less convinced; there was fear that they could not fulfill high expectations of themselves or the app demands. Second, a recurring topic in the discussion was personalization; individuals can have different preferences for exercises. Therefore, all types of exercises should be available within the app. In addition, the individual user can choose certain types of exercises. Third, some participants found it important that the choice was made in alignment with the professional caregiver. Fourth, we concretized the app’s design on 2 levels: the app’s use (VS) and its feel and look (design).

The most prominent subtopics in the use of the app were the following: when the app is used and under what conditions. Furthermore, the FG members made suggestions for more advanced use. These results are summarized in Table 6. The participants were unanimous that the best way to use OPPIs is during euthymic or mild depressive episodes. Participants were divided over the use of an OPPI in episodes of mania or hypomania. The FG members expected that use in the early stages of hypomania can provoke positive feelings and lead to a more severe manic state. However, when users are in a full manic state, they did not expect any temptation to use the app in a full manic episode because, when manic, they are quickly distracted, and the exercises require time and tranquility.

The participants also indicated that the app should be adjustable; however, most FG members think that they want to use it daily, during periods when it is beneficial to them. The duration of the practices should be between 5 and 10 minutes, so that it fits into daily routines. The participants also mentioned that allowing sufficient time and having a quiet place is important for successfully using the app. In addition, the FG members suggested that, after performing a complete set of practices, the user can choose which practices are suitable and adjust the app to those preferences. In the discussion about the potential advanced possibilities, the FG members found it helpful to connect PPIs with the Life Chart Method (LCM) [73]. A PPI should occur when an advanced set value is reached while monitoring symptoms such as mild depression (within the LCM). Incorporating PPIs in the early relapse prevention plan was also suggested, as users can apply this intervention to handle the (starting) mood episode. In the ideal world, the FG members want to be automatically directed to the PPI when they reach pre-established levels in the LCM.

During FG 2 and 3, participants were asked about their views on the *requirements* for the design of the app. The remarks obtained can be separated into the following subcategories: personalization, look and feel, text, vision and sound, and preferred options. Table 7 summarizes the results.

The participants considered personalization to be a vital aspect. The FG members wanted to have a wide range of adjustable options as long it does not affect the app’s clarity. The main topics in personalization were the frequency of use, choice between reading or listening, notifications, writing space in the exercises, and possibility to select the exercises. In addition, the proper use of wording was considered necessary by the participants. The text in the app should be concrete, clear, and short. Within the suggested exercises, the choice of words was sometimes perceived as compelling. Examples to illustrate the exercises were seen as helpful and supportive. The FG members had a preference for appealing messages in both exercises and notifications. They preferred a design that is quiet and attractive. The use of pictures can have a calming effect. We also discussed the option of obtaining feedback after completing an exercise. Approximately all participants (7/8, 88%) liked some type of feedback. They also expressed the need for external motivation to continue practicing (apart from the notifications). Significant others can be part of this motivational aspect. Valuing the exercises was seen as a good instrument to decide which exercises were preferable.

As part of the VS and design, we conducted an RPT test. Owing to technical problems in registration, 25% (2/8) of the participants did not succeed. The feedback obtained from the participants was divided into 4 categories: experienced effects, facilitating factors, impediments, and suggestions. Regarding experienced effects, 75% (6/8) of the participants found exercises to be beneficial and experienced more positive emotions than before. The experienced positive emotions did not qualify as ‘threatening’ in terms of risk for a (hypo)manic episode. One participant did not experience any difference, and another participant found it difficult to perform the exercise owing to personal circumstances. Regarding facilitating factors, the participants appreciated the layout with different pictures, videos, and music and the calm design. In addition, participants emphasized the possibility of adding the option to personalize the look and feel of the app. In total, 38% (3/8) of the participants experienced impediments while testing the app. Overall, 25% (2/8) of the participants mentioned that they were very severely depressed to perform the exercise.

**Table 5:** Valuation positive psychology interventions for use in bipolar disorder.

Theme	Positive remarks	Negative remarks	Appreciate (n=6), n (%)	Not appreciate (n=6), n (%)
Positive emotions	“Creates freedom to concentrate on your positive emotions.”	“It’s difficult to allow yourself to do what you want to do.”	6 (100)	0 (0)
Resilience	“Gives energy.” “Achieve relaxation.” “Seeking solutions that fit me rather than always must to...”	“Right wording is essential.”	6 (100)	0 (0)
Positive relations	“Focus on connecting with other people.” “The positive contact with others inspires me.”	“Contacts have to be trusted before sharing feelings.”	5 (83)	0 (0)
Strengths	“No remarks were made.”	“I don’t give myself time for that either.” “Right wording is essential.”	5 (83)	0 (0)
Self-compassion	“Allow yourself to comfort yourself.”	“It’s problematic to allow yourself to give some consolation.” “It mustn't be compulsory.” “Difficult to perform the exercises.”	4 (67)	2 (33)
Optimism and hope	“Hope is important; perhaps the exercise doesn't fit.”	“Doesn't fit people with perfectional traits.” “High expectations that maybe can’t be satisfied.” “It seems to be a bit ‘Trump-like.’” “Too ambitious.”	3 (50)	2 (33)

**Table 6:** Design and overview of the use of web-based positive psychology interventions for bipolar disorder.

Subcategories in using and remarks made by FG^a^ members			Agreed by all FG members
**When to use the app**			
	“In gloomy periods but not in severe depressive episodes.”^b^	Yes	
	“In mild hypomanic episodes.”^c^	Yes	
	“In euthymic episodes.”	Yes	
	“Suggest the user exercises on fixed times.”	No	
	“Adjustable frequency of the exercises.”	Yes	
**How to use the app**			
	“Work through all exercises; eg, 1 exercise every day for 6 weeks and then integrate it into the Life/Chart.”	Partly	
	“In the beginning, the user goes through a module with practices of the themes; positive emotions, positive relationships and resilience.” and “the themes hope, and optimism, strengths and self-compassion are offered as an option.”	Yes	
	“Suggest clearly to do the exercises in a safe environment.”	No	
	“Set up realistic goal setting.”	Yes	
	“The duration is between 5-10 minutes per exercise.”	Yes	
**Advance possibilities**			
	“Connection with the LCM^d^.”	Yes	
	“Connection with the relapse prevention plan or other recovery plans.”	Yes	
	“Going through the different exercises with the practitioner to choose a set of exercises.”	No	

^a^FG: focus group.

^b^Compared with Life Chart Method—mild or moderate depression.

^c^Compared with Life Chart Method—mild hypomanic episodes.

^d^LCM: Life-Chart Method.

**Table 7:** Design and overview of the feel and look requirements.

Subcategories and remarks made by FG^a^ members			Agreed by all FG members
**Personalization**			
	“The choice between reading or listening to the exercise.”	Yes	
	“Pleasant voice; voices can be chosen.”	Yes	
	“Space to type keywords within the exercises.”	No	
	“Ability to select which exercise you want to do.”	Yes	
	“The degree of customization must be large, but the app must remain clear to promote easy use.”	Yes	
	“The notifications must be flexible, with the option of carrying out the exercise later.”	Yes	
	“Personalization, not only in exercises but also in the used pictures, videos and music fragments.”	Yes	
**Look and feel—text**			
	“There must be a choice between spoken or written exercises.”	Yes	
	“Use not too many words; make clear short exercises.”	Yes	
	“The text should be inviting with a smooth choice of words but not too clever and easy to read.”	Yes	
	“Working with examples in the exercises.”	No	
	“Limited the amount of text per screen.”	Yes	
**Look and feel—vision**			
	“Quiet design, with a nice layout and images.”	Yes	
	“Use appropriate images for the exercises.”	Yes	
	“Offer the possibility to add pictures yourself.”	Yes	
	“Be visually appealing; photos/graphics.”	Yes	
	“Use animations for the explanation in the exercises.”	No	
**Look and feel—sound**			
	“Pleasant voice; voices can be chosen.”	Yes	
	“There must be a choice between spoken or written exercises.”	Yes	
	“Possibility to add music.”	No	
**Preferred options**			
	“Feedback, compliments after every completed exercise.”	Partly^b^	
	“Selection menu for the exercises.”	Yes	
	“Add your own exercises (in a simple layout or only as a reminder).”	No	
	“Being able to give a score yourself and make this visible in a graph.”	No	
	“To be able to share the results of the exercises with others.”	Yes	
	“Receive an anonymous response from others–as a tip or encouragement, which must be adjustable.”	No	

^a^FG: focus group.

^b^A participant did not like the option to obtain feedback in the app.

### Assessment of Use and Satisfaction With PP App and Interventions (Design and Operationalization)—Exercises of the Intervention

On the basis of the consensus reached in the FG and results of the RPT, we developed the Wellbeing Bipolar Disorder app. The app contains 7 exercises in the 4 domains of PP preferred by FG members. The exercises based on the previous work of Bohlmeijer and Hulsbergen [74] are shown in Table 8. The app differs from other apps that provide PPIs, primarily owing to the selection of PPIs. Some exercises were withdrawn because of the fear of compassion or had to be altered. Other exercises were withdrawn because they may provoke symptoms of mania. Moreover, the app had special design features, such as calm design, proper use of wording, and so on, which, according to the FG members, can lead to high compliance rates. Finally, the app can be integrated into the LCM.

**Figure 1:**
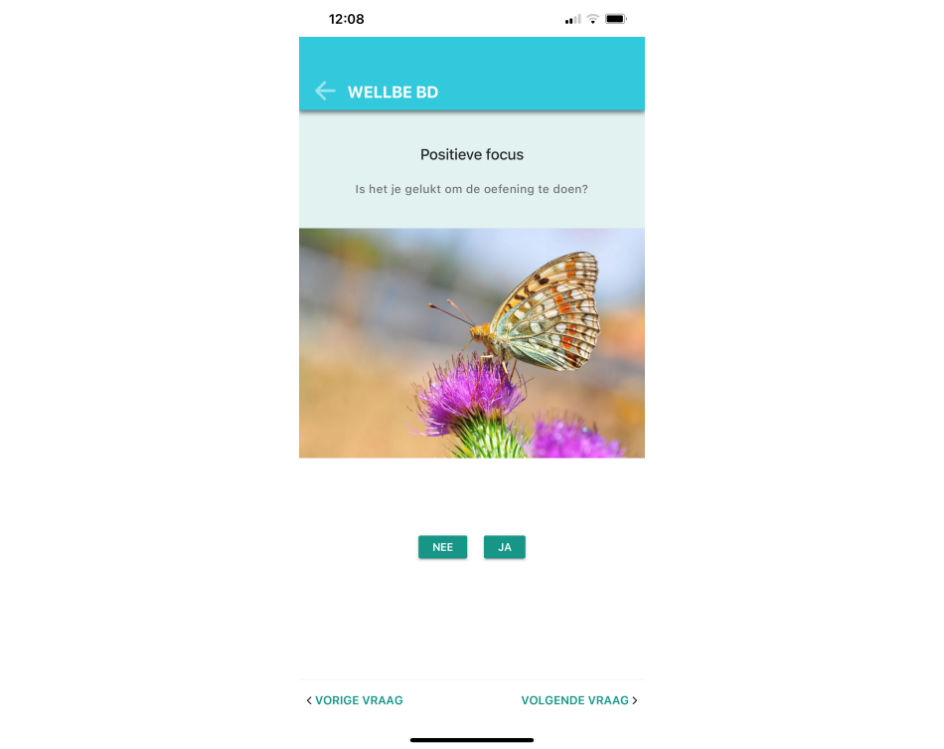
Screenshot of the intervention.

We chose a 1-week period in which the participants were provided 1 exercise daily, with mood monitoring before and after exercise and a valuation of the exercise after completing it. The app was personalized with the following choices: when to use the app, notifications, types of notifications, written or spoken video explanation, and guidance by a professional or expert by experience*.* The app’s design was calm. All exercises had 1 picture throughout the practice (Figure 1).

The participants were asked if they had succeeded in the exercise; if they did not, they could start again. We wrote 2 different scripts (professional or expert by experience), and the videos were set in the background picture belonging to the exercise.

After the development process, we tested our app to evaluate whether the results of the previous steps of the development process have been implemented to the participants’ satisfaction. The results of the PT are shown in Tables 9 and 10.

Of the 133 exercises (7×19 participants), 87 (65.4%) were completed. Patients completed more (101/133, 75.9%) exercises than professionals (72/133, 54.1%). In total, 11% (2/19) of the participants chose the video explanation. Of the 19 participants, 8 (42%) preferred the expert by experience and 8 (42%) preferred the guidance by a professional. Overall, the average rating of all exercises in total was 7.35 (scale 0-10, SD 0.525), and the median was 7.5, with a slightly high rating among professionals (mean 7.7 vs 6.9; median 7.5 vs 7.25).

The evaluation of the individual exercises was between 7.5 (exercise: be strong and be stronger) and 6.6 (exercise; listing to good news). Notably, a participant rated all exercises relatively low (average 3.3), and owing to the small sample size, this influenced the total outcome. The other individual ratings were between 8.7 and 5.6.

The app’s overall valuation was high; 91% (15/16) of the participants were positive about the app and wanted to use it for an extended period. However, according to 55% (9/16) of the participants, the frequency of the exercises seemed very high for an extended period. Besides positive comments, made in the app’s evaluation, about the effect of the exercises, there were remarks for improvement of the app. Some comments referred to the intensity of the exercises; a new exercise every day is not doable for all participants. Another advised option is to read the exercises in the morning to accomplish it during the day.

**Table 8:** Domains and exercises in the Well-being Bipolar Disorder app.

Exercise number	Exercise	Domain of positive psychology
1	Experience positive moments again	Positive emotions
2	Active listening to good news	Positive relations
3	Three good things exercise	Resilience
4	Discover your strengths	Strengths
5	Positive focus	Positive emotions
6	Expressing gratitude	Positive relations
7	Being strong and becoming stronger	Resilience

**Table 9:** Overview of the outcome of pilot test of the Well or being Bipolar Disorder app I.

Exercise number	Number accomplished^a^	Valuation by patients (scale 0-10)^b^	Valuation by professionals (scale 0-10)^c^	Valuation—total (scale 0-10), mean (SD)^d^
1	14	7.37	7.5	7.43 (0.650)
2	12	5.7	7.8	6.58 (1.054)
3	13	7.37	8.2	7.69 (0.418)
4	13	6.87	7	6.92 (0.065)
5	13	7.22	8	7.38 (0.411)
6	11	6.28	8	6.91 (0.870)
7	11	7.42	7.8	7.45 (0.211)

^a^Total number accomplished=87.

^b^Valuation by patients (total)=6.85.

^c^Valuation by professionals (total)=7.7.

^d^Valuation by all participants (total)=7.25.

**Table 10:** Overview of the outcome of pilot test of the Well-being Bipolar Disorder app II.

Participants	Percentages of accomplished exercises^a^	Valuation of the app (positive)^b^, %	More extended use of the app (positive)^c^, %	Frequency to high^d^, %
Patients	(75.7)	83	83	30
Professionals	(54)	100	100	80

^a^Number of exercises (total)=65.4%.

^b^Valuation of the app (positive; total)=91.5%.

^c^More extended use of the app (positive; total)=91.5%.

^d^Frequency to high (total)=55%, SD 0.525%.

## Discussion

### Principal Findings

This study investigated the opinions of care professionals and patients with BD regarding OPPI for BD.

The first aim was to rate the extent to which participants had experience with PPIs and expected them to be supportive in addressing unmet needs. We found that the participants did not have experience with specific evidenced PPIs; however, they mentioned experiences in adjoining therapeutic areas (eg, mindfulness) or more personal PP solutions. The FG members mentioned possible advantages: focusing on small steps, making positive pictures or movies, monitoring positive feelings, and giving yourself a positive message. These topics can be related to various unmet needs in treating BD, specifically, offering hope, increasing self-esteem, expressing feelings, promoting acceptance, and preventing social isolation. Regarding expectations about PPIs, participants expect that PPIs can accomplish some unmet needs in BD.

The second aim was to establish the preferences and requirements of the app with PPIs.

First, we determined which PP exercises patients and professionals prefer. Positive emotions, resilience, positive relations, and strengths were highly valued among the 6 categories of PP exercises. Some exercises were withdrawn due to the fear of compassion or had to be altered. Other exercises were withdrawn because they may provoke symptoms of mania. Moreover, the app had special design features, such as calm design, proper use of wording, and so on, which, according to the FG members, can lead to high compliance rates. Finally, the app can be integrated into the LCM. Therefore, we developed an app that differs from other apps that provide PPIs, primarily owing to the selection of PPIs.

The third aim of our study was to evaluate the use and satisfaction of the app. An interesting finding is that approximately all users (15/16, 91%) found it to be beneficial to perform the exercises and wanted to do it regularly. However, the frequency of the exercises seems to be very high. The valuation of the exercises was promising (7.35 on a scale of 1-10; median 7.5). Despite the small number of participants, we seemed to have found the proper exercises for our target group. Before releasing the app for clinical practice, further studies with adequate measurements (quantitative and qualitative) are necessary.

### Comparison With Previous Studies

Unmet needs can include topics such as support with loneliness, grief counseling, acceptation, social isolation, coping with others in social functioning [14,15,17], hope, expressing feelings, and increasing self-confidence in psychological functioning [12,14-17]. The positive expectations underscore the potential of PP for personal recovery and support the value of integrating PPIs into mental health care [57,58]. PPIs may help patients with resources that increase their ability to adapt and support them in personal recovery [24]. Our results are largely consistent with previous findings [19,75,76]. Mental well-being is recognized as an essential resource for the recovery from mental illness and in preventing relapse. Therefore, it is recommended to include mental well-being interventions (such as PPI) in the treatment [75,77].

Participants also raised some critical concerns about applying PPIs for BD. The FG members expressed concerns about fast and more severe changes in mood and energy when a used PPIs during manic or hypomanic episodes. Although “joy and amusement” are associated with increased manic severity, compassion—one of the key elements of PP—tends to decrease the symptoms of mania [78]. Positive emotions are not often mentioned as triggers that can provoke manic episodes. Lack of sleep is the most predominant factor in triggering manic episodes [79]. However, amorousness is the predominant factor among young adults, followed by stressful life events [80]. Periods of strong personal growth are also factors that induce manic symptoms (such as an extremely motivational workshop) [80]. Although this seems to be linked to PPIs, it is not satisfactory to conclude that PPIs can induce manic episodes. In a study among a large population of patients with BD (n=149), by applying a group PPI, the researchers found no increase in manic symptoms, thus confirming our findings [55]. In contrast, damping emotions lead to more severe depressive symptoms [81]. Therefore, it is recommended to inform potential participants properly before applying PPIs for BD. Interestingly, the PT results did not show dysregulation among the participants.

An unexpected finding was that the categories of self-compassion, optimism, and hope had low ratings. On the basis of the unmet needs, as described previously, we did not expect this outcome. This result may be explained by the fact that the FG members found the themes and exercises to be very ambitious. For example, the exercise, “the best possible self,” seems not to fit with the participants’ level of self-esteem. They had high expectations that they could not satisfy.

The “fear of compassion” can explain the fact that the FG members valued compassion relatively low. The fear of compassion is closely linked to self-criticism and depression [81]. Women with BD seem to be more self-critical than controls [82]. Nitzburg et al [83] suggest that the negative experiences in the course of BD can worsen the level of self-criticism and argue that in an early stage of the illness, interventions should also target decreasing self-criticism. However, owing to the small sample size, we cannot rule out that our findings are coincidental. Nevertheless, it is necessary to develop interventions targeting compassion and hope in BD to pay attention to the fear of compassion and transform exercises in a feasible manner for patients with BD.

We determined when to use the app. Some of the FG members expect the exercises to be a daily routine, for example, in addition to mood monitoring. Integration with a (digital) mood monitoring application and relapse prevention plan can provide a comprehensive tool in which the PPIs assist in preventing severe mood episodes [84]. The FG members foresaw the use mainly during euthymic or mild depressive episodes. The members of the FGs were unanimous in that they did not expect any beneficial effects from PPI in severe depressive episodes. In contrast, they expected the opposite effect: worsening of depressive feelings if the goals of the exercises are not achieved. Previous meta-analyses have demonstrated that PPIs seem to be more effective in populations that are nondepressed or mildly depressed [28,50] than in populations with major mood disorders [50]. This supported the FG members’ suggestion to apply PPI only in mild depressive episodes and euthymic episodes. Carr et al [85] published a meta-analysis showing that in patients with depression, PPIs had a small to moderate effect on depression in terms of symptom reduction. However, in that paper, the severity of the depression was unclear. The considerations mentioned previously can underline our finding that personalization is one of the main topics in designing a PPI app for BD. Lack of personalization is, among with depressive symptoms, an important barrier to adapting digital mental health interventions [86]. Recognizing and resolving these barriers in the development process can contribute to the adaptation of new interventions [86].

Finally, we reflected on the method. We recognized that cocreation and user involvement are important in developing an app to be accepted by end users [87]. Specific findings, such as the preferences for the exercise categories or the possible risks in applying PPIs, will not be revealed, as we developed the app without user involvement. The participation of end users is the main element in user-centered design, defined by Preece et al [88] as “an approach, which views knowledge about users and their involvement in the design process as a central concern.” The development process conformed to the CeHRes principles [70]. This method allowed us to systematically involve users (patients and professionals) in the study’s development process. This enabled us to modify the app’s design through all stages of the development process and guaranteed maximum involvement of all stakeholders. Previous studies using the same principle support our findings [88]. The development of new interventions benefits from user involvement in all stages to meet the target group’s needs [89].

### Limitations

Our study has some limitations. When recruiting professionals, there is a risk of selection bias owing to the voluntary recruitment from a highly specialized professional pool (the Dutch Foundation for Bipolar Disorders). It is conceivable that the professionals interested in web-based monitoring are stepping forward to participate in this study. However, this method was chosen to obtain professional input from different parts of the Netherlands to avoid inputs from only one region. The same applies to the consumers; they were partly recruited from an outpatient clinic and partly from the Dutch advocacy network, “Plusminus.” Although this may raise the question of whether the participants represent the target group, we assume that, by combining advocacy members and patients treated in an outpatient clinic, our study is sufficiently representative.

The use of FGs has some limitations, such as the possibility of “group effect,” in which patients tend to adapt to the group leading opinions. Therefore, it is difficult to separate a personal opinion from a group opinion [90]. We tried to avoid this bias by collecting individual data (post-it memos) before the discussion in the group. Furthermore, it is sometimes difficult to generalize the outcome of FGs [90]. We tried to avoid this bias as much as possible through nationwide recruitment.

Finally, this study is mainly a qualitative study with a relatively small number of participants; therefore, it remains uncertain whether the results are sufficiently generalizable.

Despite these limitations, we believe that we shed light on consumers’ and professionals’ thoughts and considerations about using PP apps for BD.

### Conclusions and Practical Implications

Despite recognizing the possible benefits of PPI in BD and that they may address unmet needs in BD, very little is known about the effect of applying PPI in the treatment of BD.

In this study, we realized the shared assumptions about the application of PPIs for BD. The consensus on the different topics regarding the use of PPI shows that both patients and professionals underline the beneficial possibility of applying PPIs for BD. The use during subsyndromal and mild depressive episodes seems to be the most fruitful period for patients with BD. We did not establish the risk of provoking mania or hypomania by performing PPIs, but we could not draw firm conclusions because of the small sample size.

With the systematic use of user involvement (patients and professionals) in all steps of the development process, we were able to create an app that can potentially fulfill some of the current unmet needs in the treatment of BS.

The use of PPI for BD is intriguing and can be usefully explored in further studies. We emphasize that more evaluation studies (quantitative and qualitative) that are focused on the effect of PPIs in the treatment of BD should be conducted. In addition, to establish the working mechanisms in BD, explorative, qualitative, designed studies are required to reveal whether PPIs can address unmet needs in BD.

## Abbreviations

BD: bipolar disorder
CeHRes: Center for eHealth and Disease Management
CHIME: connectedness, hope, identity, meaning, and empowerment
FG: focus group
FGD: focus group discussion
LCM: Life Chart Method
OPPI: online positive psychology intervention
PP: positive psychology
PPI: positive psychology intervention
PPT: paper prototyping
PT: pilot test
RPT: rapid prototyping
VS: value specification
